# Gradient diffusion susceptibility testing for *Neisseria gonorrhoeae*: an accurate alternative to agar dilution in high-MIC strains?

**DOI:** 10.1099/acmi.0.000116

**Published:** 2020-03-25

**Authors:** Michaël Desjardins, Brigitte Lefebvre, Christian Lavallée, Annie-Claude Labbé, Florian Mauffrey, Irene Martin, Jean Longtin, Claude Fortin

**Affiliations:** ^1^​ Department of Microbiology, Infectious Diseases and Immunology, Faculty of Medicine, Université de Montréal, Montreal, QC, Canada; ^2^​ Medical Microbiology and Infectious Diseases, Centre Hospitalier de l’Université de Montréal, Montréal, Canada; ^3^​ Laboratoire de santé publique du Québec, Montréal, Canada; ^4^​ Medical Microbiology and Infectious Diseases, Hôpital Maisonneuve-Rosemont, Montréal, Canada; ^5^​ National Microbiology Laboratory, Winnipeg, Canada

**Keywords:** *Neisseria gonorrhoeae*, antimicrobial susceptibility testing, gradient diffusion

## Abstract

**Introduction:**

The correlation of antimicrobial susceptibility testing (AST) between agar dilution and gradient diffusion for *Neisseria gonorrhoeae* is not well established, especially in strains with high MICs.

**Aim:**

The objective of this study was to evaluate the accuracy of gradient diffusion for *N. gonorrhoeae *.

**Methods:**

Fifty strains of *N. gonorrhoeae *, all tested by the agar dilution method according to CLSI methods and confirmed to be genetically distinct using molecular typing (NG-MAST), were selected. Isolates with high MICs were targeted. Gradient diffusion was performed for ceftriaxone (CRO), cefixime (CFX), azithromycin (AZT), tetracycline (TET) and fosfomycin (FOS) using two different commercial antimicrobial strips on different culture media (a non-commercial GC agar base with 1 % defined growth supplement and two commercial media). The performance of agar gradient diffusion was assessed based on accuracy, using essential and category agreements (EA and CA).

**Results:**

Essential and categorical agreement were over 90 % for CRO, CFX and AZT on the two commercial agar media tested. Category disagreements were seen for CFX and AZT, mostly just very major errors. For TET, EA ranged from 80 to 96 % and CA ranged from 38 to 76 %, most of the misclassifications being minor errors. Finally, EA for FOS ranged between 80 and 98 %.

**Conclusion:**

Gradient diffusion is an accurate and acceptable alternative for CRO, CFX and AZT. Caution is advised when MICs are reported by gradient diffusion approach breakpoints because of the possibility of very major errors. The use of gradient diffusion is limited for TET because of the high rate of minor errors.

## INTRODUCTION

According to the World Health Organization (WHO), gonorrhoea is a major public health challenge because of increasing antimicrobial resistance [1]. In a recent report on an *N. gonorrhoeae* resistance surveillance programme in Quebec, it was shown that the resistance rate for azithromycin (AZT) had increased dramatically in recent years, being 27.6 % in 2017 [2]. Moreover, the first case of *N. gonorrhoeae* non-susceptible to ceftriaxone (CRO) and to cefixime (CFX) was described in 2017 [3]. These findings are concerning because the current treatment recommendation for gonorrhoea infection consists of a third-generation cephalosporin (3GC) combined with AZT [4].

The current gold standard to testing for MIC determination in *N. gonorrhoeae* is agar dilution [1], a labour-intensive and expensive technique that is usually performed in reference laboratories. Disc diffusion is an easier, less expensive alternative to agar dilution, and is also recommended by the Clinical and Laboratory Standards Institute (CLSI). Unfortunately, it cannot be used to obtain MIC nor to test AZT because no interpretative criteria have been published by the CLSI [5, 6].

Agar gradient diffusion is an alternative to agar dilution for determination of MICs and also has the advantages of being less time-consuming and less expensive. Previous studies showed that this method was accurate when used for testing *N. gonorrhoeae* [7–9]. Most of the tested isolates were susceptible to the antibiotics currently used for treatment. For example, Shende *et al*. reported an excellent essential and category agreement (≥95 %) using the gradient diffusion method for CRO in susceptible strains [7]. However, physicians are concerned about the accuracy of this method in strains with higher MICs in light of the steady increase in *N. gonorrhoeae* resistance worldwide [10].

The primary objective of this study was to evaluate the accuracy of agar gradient diffusion compared with agar dilution for *N. gonorrhoeae* antimicrobial susceptibility testing (AST) in isolates with high MICs to the antibiotics currently used for gonorrhoea treatment (CRO, CFX and AZT). Secondary objectives were to evaluate the influence of different culture media, including commercially available ones, and different commercial antimicrobial strips on MIC results. We also aimed to evaluate the accuracy of diffusion gradient for fosfomycin (FOS), a potential alternative treatment for gonorrhoea treatment. Finally, because of previously suspected low performance in our clinical setting, disc diffusion testing for tetracycline (TET) was also assessed. The study protocol was approved by the Centre Hospitalier de l’Université de Montréal (CHUM) ethics committee.

## METHODS

### ​* Neisseria gonorrhoeae* isolates

Fifty *Neisseria gonorrhoeae* isolates were selected: 34 isolates from clinical specimens, 14 WHO reference strains and two ATCC strains (see Appendix, available in the online version of this article). The clinical isolates were carefully selected from all positive cultures in Quebec, Canada, between 2015 and 2016, according to their AST profiles. Isolates with MIC≥0.03 µg ml^−1^ to CRO or CFX or with MIC≥2 µg ml^−1^ to AZT were selected. There was no clinical information collected in relation to the selected isolates. WHO reference isolates were also chosen because of their higher MIC profiles [11]. All selected isolates were confirmed to be genetically distinct using molecular typing (NG-MAST) [12] to ensure that the same strain was not tested more than once. NG-MAST was performed at the Laboratoire de santé publique du Québec (LSPQ, Montreal, Quebec).

### ​Antimicrobial susceptibility testing

Agar dilution was performed at the LSPQ on each of the 50 isolates using the standard protocol, which is described in CLSI M07 [13]. *N. gonorrhoeae* is a fastidious organism, and it therefore needs addition of growth factors for AST. So as recommended by the CLSI, the following were added to the agar base for testing: 1.1 g l-cysteine, 0.03 g guanine HCl, 3 mg thiamine HCl, 13 mg para-aminobenzoic acid (PABA), 0.01 g B12, 0.1 g cocarboxylase, 0.25 g NAD, 1 g adenine, 10 g l-glutamine, 100 g glucose and 0.02 g iron nitrate. Moreover, when testing FOS, agar media were supplemented with glucose 6-phosphate at 25 µg ml^−1^. Interpretative criteria from CLSI M100 were used except for FOS, for which there are no published criteria [2]. The gradient diffusion AST method was performed at the CHUM on all isolates. Two different antibiotic strips (bioMérieux and Alere) were tested according to the manufacturers’ recommendations. Both techniques were performed using directly suspended colonies, adjusted to an optical density of 0.5 McFarland, from overnight cultures on chocolate agar. For gradient diffusion, the organisms were evenly spread on two different commercial media (GC agar base with 1 % GCHI; Oxoid and GC II Isovitalex; Becton Dickinson) and on the media recommended by the CLSI for AST of *N. gonorrhoeae* (GC agar base with 1 % defined growth supplement cysteine-free) which was prepared at the LSPQ [5]. Up to four strips placed in a radial fashion were applied to the 150 mm plates, which were incubated for 20–24 h at 35–37 °C in an atmosphere containing 5 % CO_2_ [14]. The agar dilution and gradient diffusion methods were compared for five antibiotics: CRO, CFX, AZT, TET and FOS. Disc diffusion testing for TET was done according to CLSI recommendations [15]. Quality control testing was performed during each day of testing for all methods using *N. gonorrhoeae* ATCC 49226 and interpreted according to the ranges published by the CLSI [2]. The study protocol was approved by the CHUM’s research ethics committee.

### ​Analysis

The accuracy of the gradient diffusion compared to agar dilution was evaluated using essential agreements (percentage of isolates with MIC within one doubling dilution) and category agreements (percentage of isolates producing the same category result: susceptible, intermediate or resistant), as recommended in CLSI M52 [16], for all different combinations of antimicrobial strips and AST media. For category disagreements, the proportions of major errors (agar dilution result is susceptible, gradient diffusion result is resistant), very major errors (agar dilution result is resistant, gradient diffusion result is susceptible) and minor errors (one result is intermediate and the other is susceptible or resistant) were calculated. The gradient diffusion method is considered an acceptable alternative to agar dilution if essential and category agreement are ≥90 %, as recommended by the CLSI [16]. For FOS, only essential agreements were calculated because no interpretative criteria have been published by the CLSI for *N. gonorrhoeae *. Reproducibility was also tested for each combination of strips and AST media. Reproducibility is defined as the closeness of agreement between the results of successive measurements of the same analyte [16]. As recommended by the CLSI, five isolates (strains 10, 15, 20, 25 and 30 – see Appendix) were tested three times each. MIC results within one doubling dilution were considered equivalent results.

## RESULTS

Results of the agar dilution MIC distribution obtained with the 50 isolates for CRO, CFX, AZT, TET and FOS are presented in Table 1. For 3GCs and for AZT, essential agreements and category agreements of gradient diffusion were all ≥90 %, except when CLSI media were used (Table 2). For TET, essential agreements ranged from 80 to 96 % and category agreements from 38 to 76 %. In comparison, disc diffusion for TET showed category agreements ranging from 22 to 38 %. Descriptions of category errors are given in Table 3. For CFX and AZT, the vast majority of errors were very major errors whereas for TET they were mostly minor errors. Reproducibility results are shown in Table 4. For the vast majority of strips/media combinations, except when CLSI media were used, reproducibility was perfect.

**Table 1.** Results of antimicrobial susceptibility testing using agar dilution (*n*=50)

	Number of isolates according to MIC (μg ml^–1^)														
	<0.03	0.03	0.06	0.12	0.25	0.5	1	2	4	8	16	32	64	128	256
Ceftriaxone*	26	7	8	5	–	2	2	–	–	–	–	–	–	–	–
Cefixime*	22	9	3	2	5	5	–	4	–	–	–	–	–	–	–
Azithromycin†	–	–	4	4	13	12	2	6	2	6	–	–	–	1	–
Tetracycline‡	–	–	–	2	5	11	9	20	–	1	2	–	–	–	–
Fosfomycin§	–	–	–	–	–	–	–	–	–	–	19	27	3	1	–

*Susceptible, ≤0.25 µg ml^−1^; non-susceptible, ≥0.5 µg ml^−1^.

†Susceptible, ≤1 µg ml^−1^; non-susceptible, ≥2 µg ml^−1^.

‡Susceptible, ≤0.25 µg ml^−1^; intermediate, 0.5–1 µg ml^−1^; resistant, ≥2 µg ml^−1^.

§No interpretative criteria have been published by the CLSI.

**Table 2.** Essential (EA) and category agreements (CA) of gradient diffusion versus agar dilution for different combinations of antimicrobial strips and culture media (*n*=50).

		Alere		bioMérieux	
		EA (%)	CA (%)	EA (%)	CA (%)
Ceftriaxone	CLSI	86	100	94	100
	BD	98	100	98	100
	Oxoid	98	100	98	100
Cefixime	CLSI	90	88	92	90
	BD	98	98	100	96
	Oxoid	98	92	94	94
Azithromycin	CLSI	82	88	80	92
	BD	90	90	94	94
	Oxoid	94	96	96	98
Tetracycline	CLSI	80	40	86	38
	BD	96	76	96	62
	Oxoid	82	52	82	38
Fosfomycin	CLSI	88	–	92	–
	BD	96	–	98	–
	Oxoid	80	–	96	–

CLSI: GC agar base with 1 % defined growth supplement

na, not applicable.

**Table 3.** Rates of minor errors (mE), major errors (ME) and very major errors (VME)

		Alere			Biomérieux		
		mE, *n* (%)	ME, *n* (%)	VME, *n* (%)	mE, *n* (%)	ME, *n* (%)	VME, *n* (%)
Ceftriaxone	CLSI	–	0/46 (0)	0/4 (0)	–	0/46 (0)	0/4 (0)
	BD	–	0/46 (0)	0/4 (0)	–	0/46 (0)	0/4 (0)
	Oxoid	–	0/46 (0)	0/4 (0)	–	0/46 (0)	0/4 (0)
Cefixime	CLSI	–	2/41 (4.9)	4/9 (44.4)	–	0/41 (0)	5/9 (55.6)
	BD	–	1/41 (2.4)	0/9 (0)	–	0/41 (0)	2/9 (22.2)
	Oxoid	–	0/41 (0)	4/9 (44.4)	–	0/41 (0)	3/9 (33.3)
Azithromycin	CLSI	–	0/35 (0)	6/15 (40)	–	1/35 (2.9)	3/15 (20)
	BD	–	0/35 (0)	5/15 (33.3)	–	0/35 (0)	3/15 (20)
	Oxoid	–	0/35 (0)	2/15 (16.7)	–	0/35 (0)	1/15 (6.7)
Tetracycline	CLSI	29/50 (58)	0/7 (0)	1/23 (4.3)	31/50 (62)	0/7 (0)	0/23 (0)
	BD	11/50 (22)	0/7 (0)	1/23 (4.3)	18/50 (36)	0/7 (0)	1/23 (4.3)
	Oxoid	24/50 (48)	0/7 (0)	0/23 (0)	30/50	0/7 (0)	1/23 (4.3)

**Table 4.** Reproducibility of gradient diffusion versus agar dilution for different combinations of antimicrobial strips and culture media

		Alere, Reproducibility, *n* (%)	Biomerieux. Reproducibility, *n* (%)
Ceftriaxone	CLSI	14/15 (93.3)	15/15 (100)
	BD	15/15 (100)	15/15 (100)
	Oxoid	14/15 (93.3)	15/15 (100)
Cefixime	CLSI	15/15 (100)	15/15 (100)
	BD	15/15 (100)	15/15 (100)
	Oxoid	15/15 (100)	14/15 (93.3)
Azithromycin	CLSI	14/15 (93.3)	13/15 (86.7)
	BD	15/15 (100)	15/15 (100)
	Oxoid	15/15 (100)	15/15 (100)
Tetracycline	CLSI	15/15 (100)	15/15 (100)
	BD	15/15 (100)	15/15 (100)
	Oxoid	15/15 (100)	15/15 (100)
Fosfomycin	CLSI	14/15 (93.3)	14/15 (93.3)
	BD	15/15 (100)	15/15 (100)
	Oxoid	15/15 (100)	15/15 (100)

Interestingly, with CFX, all the very major errors were made when MICs obtained by gradient diffusion were between 0.03 and 0.25 µg ml^−1^. For AZT, all the very major errors were made when MICs were between 0.5 and 1 µg ml^−1^. In fact, category agreement of gradient diffusion was perfect when MIC was <0.03 µg ml^−1^ for CFX and <0.5 µg ml^−1^ for AZT. These cut-offs were further validated in another clinical microbiology laboratory (Hôpital Maisonneuve-Rosemont, Montreal, Quebec). All the antimicrobial strips and media combinations were retested in this centre for CFX and AZT and the results confirmed that the cut-offs are reliable to avoid very major errors.

## DISCUSSION

Agar dilution, the gold standard to obtain MICs for *N. gonorrhoeae *, is labour-intensive and expensive. Agar gradient diffusion seems a promising alternative as it reduces laboratory workload. In our study, gradient diffusion showed an acceptable accuracy to determine MICs for *N. gonorrhoeae* in a carefully selected panel of high-MIC strains. Most of the combinations of antimicrobial strips and culture media showed essential and categorical agreements >90 %, which is considered an acceptable performance according to the CLSI [16].

An important finding of our study is that an important proportion of very major errors occurred with CFX and AZT. Papp *et al*. have previously shown that MICs using gradient diffusion for CFX were consistently underreported by one or two dilutions [8]. Liu *et al*. showed the same tendency of the agar diffusion method to produce lower MICs for 3GC [17]. We showed that this underestimation of MICs may lead to very major errors. However, we established that with MIC <0.03 µg ml^−1^ for CFX and <0.5 µg ml^−1^ for AZT the risk of category error is very unlikely. This observation was confirmed in another laboratory using the same isolates and methods. These cut-offs may be useful in laboratory settings.

Another finding of interest was the poor performance of gradient diffusion for TET. At 80 %, essential agreements were lower than for the other antibiotics tested and did not meet the CLSI criteria of >90 %. We also found a high proportion of category errors. This may be because most of the strains (90 %) have MICs around the breakpoints. Yeung *et al*. showed a good correlation between gradient diffusion and agar dilution for TET, with 98 % essential agreements but with category agreements of 85 % [18]. A more recent study showed category agreement of 62 % with TET, which is more in line with our findings [19]. Our results are also consistent with previous studies that showed that the vast majority of the disagreements were minor errors. Gradient diffusion was still more accurate than disc diffusion. For the latter, frequent minor errors [20] and weak correlation with gradient diffusion for TET [21] were previously reported.

Also, we noted that the CLSI media, made at LSPQ, seemed to be associated with lower accuracy (EA and CA) for CRO, CFX and AZT and lower precision (reproducibility) for CRO, AZT and FOS, although the differences observed were not statistically significant. These findings should be further assessed in future studies with higher statistical power.

FOS is an oral option that is being considered for the treatment of gonorrhoea [22]. However, no interpretive criteria have been published by the CLSI for *N. gonorrhoeae *, which complicates its utilization in clinical settings. However, we found that gradient diffusion appears to be an accurate method for AST with many combinations of media and strips reaching >90 % essential agreement with agar dilution. One limit of this observation is the absence of strains with high MICs to FOS.

Another limitation of this study was the relatively low number of strains tested. Our objective was to target *N. gonorrhoeae* isolates with a higher MIC profile to the currently recommended antibiotics for gonorrhoea treatment. We selected isolates with the highest MICs that circulated in Quebec between 2016 and 2017 and added the WHO reference isolates. This gave us the ability to test gradient diffusion on multiple isolates near the breakpoints. Moreover, all isolates were genetically different so that the same strain could not be tested twice. Because highly resistant strains were targeted, we felt that it was not relevant to add more susceptible strains in the study because category agreements for strains with low MICs have been well studied in the past [7, 17, 23].

### Conclusion

With the ever-increasing global threat of antimicrobial resistance in *N. gonorrhoeae,* it is paramount that microbiology laboratories have access to accurate, rapid and practical tools for MIC determination in *N. gonorrhoeae *. Our data show that gradient diffusion is an acceptable alternative for CRO, CFX and AZT. However, caution is advised when MICs reported by gradient diffusion approach breakpoints because of the possibility of very major errors. The use of gradient diffusion is also limited for TET because of the high rate of minor errors.

## Supplementary Data

Supplementary material 1
