# Supplementary material for: Gradient diffusion susceptibility testing for Neisseria gonorrhoeae: an accurate alternative to agar dilution in high-MIC strains?
Source: Access Microbiol. 2020 Mar 25;2(5):acmi000116. doi: 10.1099/acmi.0.000116 (PMC7494190; doi:10.1099/acmi.0.000116)
Supplement: Supplementary material 1 [file acmi-2-116-s001.pdf]

Appendix – Selection of *Neisseria gonorrhoeae* strains with their antimicrobial susceptibility testing using agar dilution and NG-MAST results

**Strain #**	**Agar dilution** (µg/mL)					**NG-MAST**
	**Ceftriaxone**	**Cefixime**	**Azithromycin**	**Tetracycline**	**Fosfomycin**	
ATCC43069	≤0.001	0.004	0.12	0.25	16	ST-681
ATCC49226	0.016	0.03	0.5	0.5	32	ST-1572
3	0.008	0.008	4	0.5	32	ST-11697
4	0.12	0.5	0.5	2	32	ST-2958
5	0.002	0.008	8	0.5	16	ST-11811
6	0.06	0.25	0.5	1	32	ST-11417
7	0.008	0.016	0.06	0.5	64	ST-7638
8	0.008	0.016	8	0.5	16	ST-9047
9	0.016	0.03	2	2	32	ST-11844
10	0.06	0.06	0.5	2	64	ST-2400
11	0.06	0.5	0.25	1	32	ST-5308
12	0.12	0.12	0.25	2	32	ST-1790
13	0.06	0.5	0.25	1	32	ST-7554
14	0.06	0.25	0.25	2	32	ST-6200
15	0.008	0.016	8	1	16	ST-5961
16	0.016	0.016	2	2	16	ST-12302

17	0.002	0.008	8	0.5	16	ST-10567
18	0.008	0.016	0.25	0.25	16	ST-9160/5441
19	0.004	0.016	0.12	0.5	128	ST-3577
20	0.004	0.008	8	0.5	16	ST-12670
21	0.06	0.12	0.25	1	16	ST-2318
22	0.016	0.016	2	2	16	ST-13814
23	0.03	0.25	0.25	2	32	ST-9991
24	0.016	0.06	1	2	32	ST-10451
25	0.002	0.008	0.2.5	0.5	32	ST-6175
26	0.03	0.03	2	1	32	ST-14030
27	0.016	0.03	0.06	0.25	32	ST-25
28	0.03	0.06	0.25	0.5	16	ST-14138
29	0.03	0.03	2	2	32	ST-14512
30	0.002	0.004	0.12	0.12	16	ST-4669
31	0.03	0.03	0.5	2	16	ST-4851
32	0.004	0.008	0.06	0.5	16	ST-2
33	0.12	0.5	0.25	1	16	ST-13876
34	0.004	0.008	0.12	0.25	16	ST-7200
35	0.004	0.016	0.06	0.12	16	ST-8118
36	0.5	>1	0.5	2	32	ST-1614
WHO F	≤0.001	0.002	0.25	0.25	32	ST-3303

WHO G	0.03	0.03	0.25	16	32	ST-621
WHO K	0.12	0.5	0.5	2	16	ST-1424
WHO L	0.12	0.25	0.5	2	16	ST-1422
WHO M	0.016	0.016	0.5	2	32	ST-3304
WHO N	0.016	0.016	0.25	16	32	ST-556
WHO O	0.06	0.03	0.5	8	64	ST-495
WHO P	0.008	0.016	4	1	32	ST-3305
WHO U	0.002	0.004	8	1	32	ST-2382
WHO V	0.03	0.03	>64	2	32	ST-8927
WHO W	0.06	0.25	0.5	2	32	ST-835
WHO X	>0.5	>1	0.5	2	32	ST-4220
WHO Y	>0.5	>1	1	2	32	ST-1407
WHO Z	0.5	>1	2	2	32	ST-4015
